# Empowering Communities through Citizen Science: Dengue Prevention in Córdoba

**DOI:** 10.3390/biology13100826

**Published:** 2024-10-15

**Authors:** Elizabet L. Estallo, Magali Isabel Madelon, Elisabet M. Benítez, Doriam Camacho-Rodríguez, Mía E. Martín, Anna M. Stewart-Ibarra, Francisco F. Ludueña-Almeida

**Affiliations:** 1 Instituto de Investigaciones Biológicas y Tecnológicas (IIBYT), CONICET—Universidad Nacional de Córdoba, Centro de Investigaciones Entomológicas de Córdoba, Facultad de Ciencias Exactas, Físicas y Naturales, Universidad Nacional de Córdoba, Av. Vélez Sarsfield 1611, Ciudad Universitaria, Córdoba X5016GCA, Argentina; elizabet.estallo@mi.unc.edu.ar (E.L.E.); benitez.elisabet@gmail.com (E.M.B.); francisco.luduena.almeida@unc.edu.ar (F.F.L.-A.); 2 Instituto Jesús María, Hipólito Vieytes 1635, Córdoba X5000, Argentina; magali.madelon@gmail.com; 3 Facultad de Enfermería, Universidad Cooperativa de Colombia Campus Santa Marta, Troncal del Caribe Sector Mamatoco, Santa Marta 470002, Colombia; doriam.camacho@ucc.edu.co; 4 Inter-American Institute for Global Change Research (IAI), Edificio104, Calle Luis Bonilla, Ciudad del Saber, Clayton 0843-0308, Panama; anna.stewart@dir.iai.int; 5 Cátedra de Matemática (Cs. Biológicas), Facultad de Ciencias Exactas, Físicas y Naturales, Universidad Nacional de Córdoba, Av. Vélez Sarsfield 1611, Ciudad Universitaria, Córdoba X5016GCA, Argentina

**Keywords:** dengue prevention, vector-borne disease, community perception, temperate, Argentina

## Abstract

**Simple Summary:**

Dengue fever, a mosquito-borne disease, is spreading more widely, and traditional methods to control the vector mosquitoes have been ineffective. In Córdoba, Argentina, we studied if involving school students in educational projects could help communities better understand dengue and take steps to prevent it. Working with students and their families showed us that people learned more about the disease and adopted better practices to stop the mosquitoes from breeding. Student engagement was a powerful way to bring this knowledge into homes, leading to positive behavior changes. This research suggests that including these programs into school curricula all year round might help fight dengue in other areas facing similar challenges.

**Abstract:**

Traditional mosquito vector control methods have proved ineffective in controlling the spread of dengue fever. This study aimed to assess the effectiveness of community engagement through student-led science in promoting dengue prevention and socioecological factors in the temperate urban city of Córdoba, Argentina. It assesses community perceptions, knowledge, attitudes, and preventive practices regarding dengue fever and its vector. Results showed a significant increase in knowledge about the vector and the disease and respondents’ adoption of good preventive practices. Student-led science was identified as a valuable tool for reaching households and leading to behavior changes at home. Furthermore, the findings highlighted the need for school programs to address vector biology and vector-borne disease prevention all year round. This study provides invaluable insights into the effectiveness of community engagement through student-led science to promote dengue prevention and socioecological factors. The findings suggest that this approach could be used to control the spread in other regions affected by the disease.

## 1. Introduction

In recent years, the Americas have faced a major dengue fever (DEN) epidemic [1]. In particular, Argentina has faced recurring challenges with the dengue virus (DENV), the only arbovirus in the country with epidemic potential. Dengue was eradicated in the country in the mid-20th century, mainly through successful control programs targeting *Aedes aegypti*, the primary vector. However, the first modern local transmission of DEN was reported in 1997, followed by an outbreak in the subtropical northern region of Argentina [2]. Since then, DEN transmission has been detected almost every year in the northernmost provinces [3]. In 2009, local DEN transmission was detected in temperate central Argentina for the first time, leading to a significantly higher incidence across most provinces [4]. Towards the end of 2015 and the beginning of 2016, the outbreak exceeded previous records: 49% more cases than in 2009 [5]. Since 2009, the number of cases has increased exponentially, leading to an extreme situation by 2024. Argentina’s most significant DEN outbreak was between epidemiology week 31/2023 and week 24/2024, with 529,625 cases reported. This number amounts to 3.18 times more cases than the same period in the previous season (2022/2023) and 7.96 times more than the same period in 2019/2020 [6].

Along with the resurgence of DEN, demographic shifts and inadequate basic urban infrastructure [7], an increase in solid waste—such as plastic containers, car tires, plastic bags, and other objects—can provide larval mosquito habitats and become a risk factor when DENV is circulating [8,9]. These risk factors and the right environmental conditions increase the likelihood of viral transmission [8].

In response, the integrated management strategy (IMS) to improve DEN control includes integrated vector and disease surveillance, vector control, community engagement, and intra- and inter-sectoral collaboration. It also supports activities like capacity building, research, advocacy, and policies and laws [10]. Several studies suggest focusing on education addressing the IMS within the ecological community. In temperate areas like Córdoba, education on vector-borne diseases increases during high vector activity, unlike in some tropical countries where DEN is active yearly. The IMS seeks to modify the behavior of individuals and the community to reduce transmission risk factors with coordinated actions within and outside the health sector [11]. Indeed, evidence suggests that education can lead to behavior changes related to DEN, as knowledge scores increased significantly after health education programs, barring a language communication gap [12].

Therefore, community education is a key element of the IMS, as it has driven significant behavior changes in dengue prevention. Previous initiatives in Honduras [13], Mexico [14], and Colombia [15] have shown the effectiveness of engaging students and their families in reducing mosquito breeding sites and promoting preventive practices at home. In Argentina, similar educational actions have been implemented in Buenos Aires, such as workshops and placing ovitraps, thus emphasizing the role of students as agents of change at home and in their communities [16].

Community engagement through “student-led science” leads to a change in attitudes at home because students become efficient health educators. This study aims to assess household perceptions, knowledge, attitudes, preventive practices, as well as motivate students to take action regarding dengue fever in Córdoba City during a DEN outbreak. This study seeks to provide further evidence on the potential for educational programs to enhance dengue prevention in temperate regions like Córdoba by examining socioecological factors and community engagement through student initiatives.

## 2. Materials and Methods

### 2.1. Study Area

**Figure 1:**
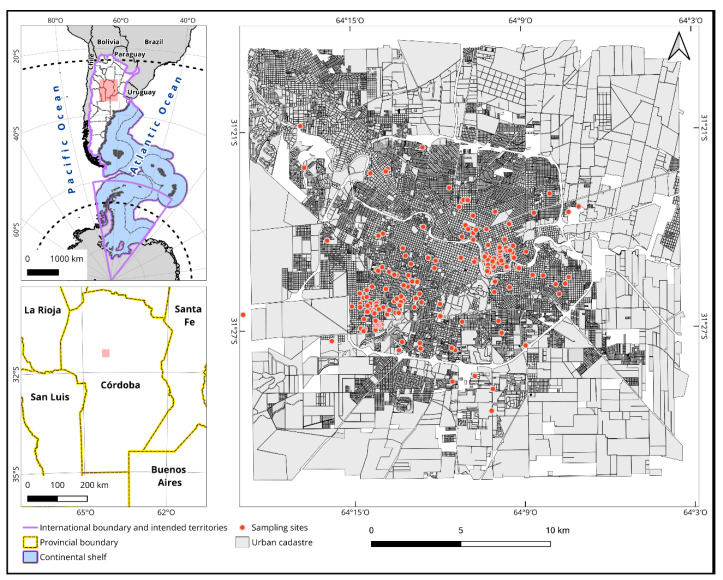
Study area. The area of Córdoba City (Argentina) where the surveys were conducted in 2020. The dots show the approximate location of the respondents, and the colors indicate the school attended by the students who conducted the surveys.

Córdoba City is located in central Argentina (Figure 1), has an area of 576 km^2^, and is home to 1,565,112 inhabitants [17]. Córdoba has a warm temperate climate featuring hot summers and four distinct seasons. It has an annual rainfall of 800 mm and an average temperature of 18 °C [18]. These climatic conditions, particularly during the rainy summer months, contribute to the creation of mosquito breeding sites, which raises the risk of dengue virus (DENV) transmission. Dengue fever was first recorded in Córdoba in 2009, causing public health concern and prompting enhanced vector control actions [19].

The National Health Ministry oversees vector control measures, and mainly involves focal control action on reported DEN cases, such as indoor and outdoor fumigation, larvicides with *Bacillus thuringiensis israelensis* (BTI), and removing standing water [20]. Public education campaigns have aimed to prevent DENV transmission, although some confusion persists regarding the distinction between the mosquito, the disease, and the virus.

In 2014, the Government of Córdoba implemented the Strategic Plan for Comprehensive Prevention and Control of DENV and Chikungunya in Córdoba [21]. This plan emphasized the need to enhance education on DEN and chikungunya across various educational levels. However, no formal educational initiatives engaging students and the community currently aim to induce behavior changes to reduce disease risk.

### 2.2. Study Design and Population

This study applied a contributory citizen science model, recruiting final-year high school students as ‘citizen scientists’ to engage their communities. This approach was selected for two reasons: it expands public health education and enables the collection of extensive household data on dengue perceptions and practices [22,23,24]. Inclusion criteria: interviewing a family member of legal age with the cognitive capacity to answer the questions. Of the 240 students initially recruited, 52 were excluded because they had interviewed minors, leaving a final sample of 188 students. This exclusion was based on ethical requirements that mandated all interviewees be adults with full cognitive capacity to provide informed responses.

The project was communicated to the principals and four natural sciences teachers from the relevant institutions to ensure unified criteria. After that, each teacher communicated the project virtually to the participating students, who were taught what mosquito breeding sites were and were shown the most common breeding sites in the city, such as cans, jars, vases, buckets, containers, pet water bowls, unused tires, toys, bottle caps, folded bags or folded canvas, pot plates, cache pots that accumulate water on the surface, drains with standing water, any unused container exposed to rain [25,26]. They were also taught about the life cycle of the mosquito and watched a YouTube video (https://www.lavoz.com.ar/videos/dengue-lo-que-tenes-que-saber-para-prevenir-enfermedad/, accessed on 15 April 2020) on mosquito habitats. Prevention and disease concepts were analyzed with them. In addition, they read a local newspaper article with an interview conducted by expert researchers in Córdoba City [27].

The research team included researchers with extensive experience in the subject and doctoral training in biological sciences (EE, FL, and EB), ecology (AS), and nursing (DC), as well as biology PhD candidates (MM and MEM).

### 2.3. Field Data Collection

The program to develop community participation in schools through student-led science was entitled “Dale Block al Dengue” (“Let’s Block Dengue”), emulating the social media concept of blocking someone when you dislike what they post and applying this to the mosquito as a DENV vector, knowing that DENV circulation can be blocked if mosquito breeding sites are eliminated [28]. The project was conducted in three phases:

- Phase 1. Household survey on perceptions and knowledge about dengue, prevention, and socioecological factors;
- Phase 2. Backyard mosquito habitat assessment;
- Phase 3. Active mosquito breeding site elimination.

Phase 1. Household survey on perceptions and knowledge about dengue, prevention, and socioecological factors

The students conducted the survey at home. The survey was created in Google Forms and made available in the Google Classroom platform, to which the students had access.

Following the established protocol, each student surveyed a family member over 18 who lived with them.

The survey was designed based on previous local field experience and years of collaboration with the Ministry of Health of Córdoba in the DEN program [29].

The information collection instrument included variables such as identifying vector-borne diseases, socioecological factors, knowledge and perception about DEN, vector prevention and control measures at home, and the home’s environmental surroundings.

The survey included 34 questions: 8 open questions and 26 closed dichotomous or multiple-choice questions (Table S1 in Supplementary Materials).

The socioecological factors included sociodemographic characteristics such as age, highest level of education, relationship with the student conducting the survey, previous DEN infections in household members or knowledge of someone infected, people living in the household, and travel to neighboring countries or any other country with vector transmission.

Knowledge questions focused on vector-borne diseases, symptoms, and knowledge about *Ae. aegypti* vectors. Participants were also asked about their perception of DEN risk, of preventive responsibility, and about how they control factors at home.

The survey included questions about the environment around the home to better report the proximity to potential vector breeding sites within 500 m of the households. This radius was selected because studies have shown that female *Ae. aegypti* mosquitoes generally fly between 100 and 500 m [30]. We formulated hypotheses for each survey category (Table 1).

Phase 2: Backyard mosquito habitat assessment

Students explored their backyards, gardens, and indoor spaces under guidance to identify potential mosquito breeding sites. They documented their findings by taking photographs of their backyards/gardens to show the presence or absence of potential breeding sites; they also photographed containers with or without water, along with the stages of mosquitoes found inside. They had to submit the photographs to their classroom teacher for certification.

Phase 3. Active mosquito breeding site elimination

Students cleaned up their backyards and gardens, removed unused artificial containers (potential habitats), and poured hypochlorite on drains they found with water (Additional File S1 in Supplementary Materials). They submitted the photographs through Google Classroom.

The activity was evaluated in the virtual classroom, where students answered the open interview question “How have your perception and practices regarding DEN control changed after participating in the ‘Block Dengue’ project?”.

This type of question explores topics in depth [31], and participants can develop their ideas through narrative competence [32]. The answers to each question become the input for analyzing qualitative information.

### 2.4. Data Analysis

Descriptive statistics were used to analyze the quantitative results of the survey through frequencies and percentages. We considered Adequate Responses about Symptoms (ARS), Adequate Responses about DEN and Vector Knowledge (ARK), and Educational Level (EL) coding and grouping some survey responses.

Relationships between ARS and EL were analyzed; ARS was worth 1 point when the respondent answered correctly and 0 points if not. We tested for the association between ARS and the respondent’s EL using logistic regression analysis. For ARK, related to the question about how dengue is spread, the answers were scored as follows: 2 points if they mentioned “through a mosquito bite”, or “that mosquito should be previously infected”, and 5 points if they included the mosquito’s scientific name, and 10 points when they explained that “it is the female that bites”.

A differentiated score was also applied to the answers to what *Ae. aegypti* is. We assigned 2 points to answers that only mentioned that it was a mosquito or a vector or that it transmitted DEN. We assigned 4 points when they said *Ae. aegypti* is the mosquito’s scientific name and that it transmits other diseases, or specified visible features of the species.

Finally, the points were added up and scores between 10 and 28 were considered to indicate sufficient knowledge. Scores under 10 were considered to indicate insufficient knowledge. These results were analyzed according to the respondent’s age and reported educational level.

In the preventive practices category, respondents were assigned scores based on actions taken to control mosquito breeding at home [33]. For example, physical removal of habitats (e.g., covering containers or disposing of unused items) was weighted most heavily, with 10 points. Other actions, like the use of insect repellent or pool chemicals, received 5 points, and lawn mowing was weighted at 1 point. Scores of 12 or higher were considered sufficient to indicate effective preventive measures.

Therefore, the measures reported by the respondents were assigned different values according to the following formula: V = 10R + 5B + 5P + 2F + S.

Where V is the score awarded to the respondent according to the actions taken, those are represented with a letter. If they are carried out, they are worth 1 point, and if they are not, they are assigned 0. R indicates acting on containers (physical removal of habitats, covering containers, placing artificial containers to reduce breeding sites). This was assigned 10 points because it is the main action against *Ae. aegypti*. B indicates protection against mosquito bites using insecticides such as repellents and coils; this was assigned 5 points. P indicates using chemicals in swimming pools: this was assigned 5 points. F indicates fumigating the dwelling and peri-dwelling with insecticides, worth 2 points. This practice eliminates mosquitoes; however, it impacts other insects and human and pet health. S indicates lawn mowing, worth 1 point because it reduces potential adult mosquito shelter sites. Therefore, the value of V can range from 0 to 23, and the actions were considered sufficient if they had a score equal to or greater than 12 points and insufficient if they had fewer than 12 points.

Preventive measures were associated with age and educational level using logistic regression analysis. All statistical analyses were performed with Infostat software (version 2020) [34].

Qualitative data were analyzed in Spanish following the thematic analysis method of textual content [35]. This resulted in two categories discussed with the research team.

### 2.5. Ethics

The Ethical Committee of the Universidad Nacional de Córdoba and the Hospital Nacional de Clínicas approved this study. Informed consent was obtained from each school principal, who acted as the formal gatekeepers for the participating students. Additionally, participants were fully informed about the study’s goals and procedures before agreeing to participate.

## 3. Results

### 3.1. Socioecological Factors

The questionnaire was completed by 188 participants, mostly aged 30+ (88.2%), living in four-person households, and having completed secondary school. Despite the major DEN outbreak, over 90% were unaware of cases in their neighborhoods. Travel outside the country, primarily to neighboring countries, peaked in January–February (summer vacation).

Hypothesis 1 (high awareness of local DEN cases) was disproved, indicating a significant gap in community awareness, despite the major outbreak. On the other hand, Hypothesis 2 (travel to risk areas in summer) was confirmed, suggesting that travel remains a key risk factor for DEN exposure (Table S2 in Supplementary Materials).

Most respondents (75%) knew that mosquitoes transmit DEN, and while symptoms such as fever and body aches were frequently mentioned, a gap in deeper knowledge persisted (Table S3 in Supplementary Materials). For instance, fewer than 15% of participants identified *Aedes aegypti* as the main vector, and only 3% knew that only female mosquitoes transmit the disease.

Television and the internet were the primary sources of knowledge, which emphasizes the need for relevant school programs (Table S4 in Supplementary Materials).

Concerning familiarity with *Ae. aegypti*, most respondents correctly identified it as a mosquito (72.68%), and over half indicated it as a DEN vector (54.12%). Some respondents identified it as a vector for other diseases (n = 14), as a specific mosquito species (n = 16), or by its scientific name (n = 19). Most participants (90.20%) knew that not all biting mosquitoes are *Ae. aegypti*, and one respondent mentioned the “common mosquito” likely referring to *Culex quinquefasciatus*.

Based on Hypothesis 2, we found that secondary school graduates usually correctly identified DEN symptoms more than expected (χ^2^ = 21.63, *p* < 0.001), supporting the hypothesis. However, there were no significant differences in knowledge about DEN and preventive practices based on educational level and age (Table 2). However, a large number of respondents (84.5%) reported good preventive practices.

### 3.2. Perception of Dengue Risk

Perception of disease severity is crucial in encouraging preventive actions. In this study, 56% of respondents considered DEN a severe disease, while 59% believed that prevention was relatively easy. These findings suggest that although there is awareness of the disease’s severity, additional efforts may be needed to address the prevention challenges perceived.

### 3.3. Household Control Practices

Most respondents (90%) reported removing stagnant water containers, a key preventive measure, while 92% used repellents to avoid mosquito bites. However, more structural solutions, such as window and door screens, were less frequent: only 24% of homes had window screens and 9%, door screens. Respondents also reported additional measures like organizing and cleaning surroundings, disinfecting their home, and using insecticides. Specifically, they cleaned water-holding containers, and gutters, and checked drains and garbage bags regularly to prevent water accumulation.

### 3.4. The Home’s Environmental Surroundings

Environmental risk factors, such as informal waste and proximity to water channels and the Suquía River, were reported by a significant number of respondents (36% and 31%, respectively). These factors, coupled with frequent mosquito sightings (88% reported daily sightings during the warm season), highlight the challenges in controlling mosquito habitats in urban areas.

### 3.5. Attitude Activity

Although 94 students (39%) completed the activity and successfully identified mosquito breeding sites, the low participation rate may have limited the scope of insights. Nevertheless, those who participated were strongly aware of potential breeding sites at home. They successfully identified mosquito breeding sites such as pots, buckets, bottles, drinking containers, jars, and vases. During the inspection, 89% of students found water inside the containers. Among them, 17% detected mosquito larvae. However, it was not possible to determine whether these were *Ae. aegypti* larvae.

### 3.6. Taking Action

Following their education, students applied their newfound knowledge by removing containers, emptying them, and storing them indoors. They documented these actions with photographs. Students reported that the project had a positive influence on their families, with 65% noting that they monitored potential mosquito breeding sites at home more closely. This suggests that student-led initiatives could catalyze broader community engagement in dengue prevention efforts. Two categories emerged from the analysis of qualitative data:

Category 1: Positive assessment

Most students positively assessed the action and mentioned that they are now more aware of mosquito breeding sites at home and the measures to prevent and eliminate them, such as cleaning containers with stagnant water and keeping spaces clean.

The information helps us prevent the spread of the disease and raise people’s awareness of the need to clean their spaces to prevent mosquito breeding, and with that, the disease (Mafalda, 17).

The action I took seemed a very good idea for me to learn and get information about the mosquitoes that spread dengue, their breeding sites, and how to identify and avoid them (Manolo, 17).

I found it very interesting … I was able to learn many more things about the mosquito that carries dengue, what the symptoms are like and how to prevent it (Susanita, 17).

These actions allowed me to learn much more about this disease and how to prevent it. In my opinion, we should all implement our newly acquired knowledge right now to combat dengue (Raquel, 17).

Category 2. Personal and family impact

Students considered that the information is crucial and should be disseminated more widely to educate the population about the importance of preventing DEN. Some suggest that everyone should implement what they have learned to combat the disease and that this action has had a positive impact on them and their families.

I think the activity is good because with this new information, for example, in my case, my brother knows more about dengue and its prevention, as do my parents when I tell them about the information from the video and the news (Felipe, 17).

I found these activities very interesting and interactive, as well as informative; all this information will make me more cautious (Paca, 17).

The activity taught us and helped us become aware of dengue (Muriel, 17).

It provided me with a lot of information about things I had no idea about, especially prevention, since we are not careful enough (Libertad, 17).

## 4. Discussion

Student-led science engaged teachers and students during online classes and the largest DEN outbreak in the Americas. According to the World Health Organization (WHO) [36], sustained community engagement can significantly improve vector management efforts. Mosquito education is implemented in temperate areas of Argentina during months when mosquitoes are active. However, these efforts are not sustained consistently throughout the year as might be in tropical regions where DEN risk is present year round.

The hypothesis about high awareness of local DEN cases was disproved, indicating a significant gap in community awareness. The second hypothesis (travel to risk areas in summer) was confirmed, suggesting that travel remains a key risk factor for DEN exposure. Most people in the study area travel to neighboring countries such as Brazil, which reported over 2,226,900 DEN cases in 2019 [37]. This increases the risk of imported cases becoming a major cause of the spread of the disease [38].

Our findings revealed a lack of knowledge about *Aedes aegypti* as the primary vector for dengue transmission. This gap in awareness mirrors the results from a study in Colombia, where lower educational levels were associated with reduced understanding of disease transmission and control practices [39]. In contrast, a study in Sri Lanka found that middle-class families with moderate education levels actively engaged in environmental cleaning, reducing the risk of mosquito breeding [40]. These contrasts highlight the need for tailored educational interventions based on specific community characteristics.

The mosquito vector seems to be better known from television and less from school, indicating that television is an essential source of knowledge, as other studies claim [41,42]. This could highlight the need for schools to include content on mosquito biology and vector-borne diseases in their curricula to raise awareness about preventing the formation of vector breeding sites and disease transmission, as suggested by the WHO.

Regarding the perception of DEN, in Córdoba, interviewees recognize the importance and severity of the disease. These findings highlight a difference compared to other countries such as Colombia, where people think that it is not a disease, but just a common flu or cold [42]. In Malaysia, most respondents recognized common symptoms or signs of the disease, but not its potential complications [41]. A study in China found that most community residents knew that DEN is transmitted by mosquitoes, that one of the symptoms is fever, and that mosquito larvae live in water; however, most were unaware of symptoms such as rashes and bleeding [43]. In tropical areas, people are used to having a large number of DEN cases each year, unlike in the temperate city of Córdoba, where vector activity occurs during the warmer months. This is why people may underestimate its importance.

Other studies highlight how responsibility for dengue control was often attributed to the government [42]. However, our study reveals a strong sense of individual responsibility among respondents in Córdoba. This suggests that local public health interventions could benefit from reinforcing the role of personal preventive measures, alongside community and government efforts. Our findings suggest that schools are essential in raising awareness about mosquito biology and prevention strategies, complementing the efforts of government-led public health campaigns. While the government is primarily responsible for providing information, fumigation, and vector control, schools are uniquely positioned to instill long-term preventive behaviors in students and their families. This dual approach—government action combined with educational initiatives—embodies the concept of ‘joint work’ and ‘social responsibility’ that many participants mentioned. All the prevention methods mentioned are recommendations from the media that highlight their relevance.

Prevention and control of DEN outbreaks requires community involvement in mosquito control through information and communication campaigns on different media and led by health personnel [43]. The results can help promote the implementation of DEN prevention and control strategies because they can be positively reinforced by schools. In the Philippines, most respondents correctly identified DEN prevention and control measures [44].

It is necessary to improve community awareness and knowledge of DEN prevention, solid waste management, and living in a clean environment, as well as vector reproducibility, mosquito bites, symptoms, and treatment [45]. Community participation is essential when implementing DEN control programs, as people’s knowledge of aspects such as vector reproduction, solid waste management, as well as signs, symptoms, and treatment strategies for DEN must be improved to obtain timely medical care and reduce the risk of death [46]. Storage of solid waste for more than seven days favors the reproduction of *Aedes* mosquitoes. Therefore, the separation of waste at home and disposal by local authorities can help curb transmission [40]. The control methods currently available target the *Aedes* mosquito, so it is necessary to implement them with community participation [47]. The participation of young people in DEN prevention can make them key actors in this fight, also involving the community, local authorities, the educational sector and religious leaders, among others [48].

## 5. Conclusions

The student-led approach in this study highlights the potential for educational programs to drive significant changes in household behaviors, particularly in eliminating mosquito breeding sites. Future dengue prevention efforts should focus on integrating mosquito biology and vector control into school curricula, not just during outbreaks, but year round, ensuring that these preventive habits are sustained over time.

Since DEN is related to health behavior, future prevention and control programs can be developed to encourage communities to make effective decisions and behavior changes regarding DEN and its prevention. Positive attitudes must be instilled and better preventive practices must be encouraged to eliminate breeding sites for mosquito vectors and thus reduce the possibility of DEN circulation in the city.

These findings underscore the critical role of community education and engagement in dengue prevention. By leveraging schools as hubs for public health education and promoting sustained individual responsibility, communities can more effectively manage mosquito populations and reduce the risk of dengue transmission. Future research should explore the long-term impact of student-led initiatives and assess how similar approaches could be adapted to other vector-borne diseases.

## Figures and Tables

**Table 1:** Hypothesis to test based on the questionnaire on vector-borne diseases, socioecological factors, knowledge and perception of dengue, prevention and vector control actions at home, and the home’s environmental surroundings.

Survey Section	Hypothesis to Test
Socioecological factor	- A large percentage of those surveyed knew of dengue cases in their neighborhoods. - Most trips to risk areas are made during summer vacations.
Knowledge about vector-borne diseases	- Having the right knowledge about the symptoms of dengue disease depends on educational level. - The main sources of information about *Ae. aegypti* and the diseases it transmits are mass media rather than formal educational institutions.
Perception of dengue, household vector prevention and control practices	- Dengue is perceived as a moderate-risk disease. - Dengue prevention is simple, and implementing it depends on each individual. - Eliminating containers that are potential breeding sites is the most frequent practice. - The use of repellents to stop mosquitoes is not frequent.
Home’s environmental surrounding	- Knowing the conditions of the surrounding environment could improve prevention attitudes. - Awareness of proximity to abandoned areas or rivers could lead to preventive measures.

**Table 2:** Knowledge about *Aedes aegypti*, dengue, and the main symptoms.

		Sufficient		Insufficient		χ^2^	*p*-Value
		n	%	n	%		
Age group	18–30 years	8	36.4	14	63.6	0.11	0.7432
	>30 years	66	40	99	60		
Educational level	Secondary	27	40.3	40	59.7	0.02	0.8794
	Higher	47	39.2	73	60.8		
Total		74	39.6	113	60.4	4.12	0.0423

n: number of respondents in each category; %: observed percentage of respondents belonging to each category; χ^2^: comparison of observed and expected frequencies in each category. Sufficient and insufficient refers to knowledge. Sufficient: respondents who received between 10 and 28 points. Insufficient: those who received less than 10 points.

## Data Availability

The data presented in this study are available upon request from the corresponding author.

## Supplementary Materials

The following supporting information can be downloaded at: https://www.mdpi.com/article/10.3390/biology13100826/s1, Table S1: Survey instrument including questions on the identification of vector-borne diseases, socioecological factors, knowledge, and perception of dengue, household vector prevention and control actions, as well as the household environmental surrounding; Table S2: Information provided by respondents on socioecological factors; Table S3: Information provided by respondents on vector-borne diseases knowledge; Table S4: Information provided by respondents on what they know about dengue and *Aedes aegypti*; Additional File S1: Additional information about teaching students.

## References

[1] Pan American Health Organization (PAHO) (2020). Actualización Epidemiológica Dengue. https://iris.paho.org/bitstream/handle/10665.2/51892/EpiUpdate7February2020_eng.pdf?sequence=1&isAllowed=y.

[2] Martino O., Weissenbacher M. (2017). Historia natural de enfermedades emergentes y reemergentes en la Argentina: Zika, chikungunya y dengue (2016–2017). Prensa Méd. Argent.

[3] Vezzani D., Carbajo A.E. (2008). Aedes aegypti, Aedes albopictus, and dengue in Argentina: Current knowledge and future directions. Mem. Inst. Oswaldo Cruz.

[4] Robert M.A., Stewart-Ibarra A.M., Estallo E.L. (2020). Climate change and viral emergence: Evidence from Aedes-borne arboviruses. Curr. Opin. Virol..

[5] Bolzan A., Insua I., Pamparana C., Giner M.C., Medina A., Zucchino B. (2019). Dinámica y caracterización epidemiológica del brote de dengue en Argentina año 2016: El caso de la Provincia de Buenos Aires. Rev. Chil. Infectol..

[6] Ministerio de Salud de la República Argentina (2024). Boletín Epidemiológico Nacional N° 709, SE 24. https://www.argentina.gob.ar/sites/default/files/2024/04/ben-709-se24.pdf.

[7] Kolimenakis A., Heinz S., Wilson M.L., Winkler V., Yakob L., Michaelakis A., Papachristos D., Richardson C., Horstick O. (2021). The role of urbanisation in the spread of Aedes mosquitoes and the diseases they transmit—A systematic review. PLOS Neglected Trop. Dis..

[8] Estallo E.L., Sangermano F., Grech M., Ludueña-Almeida F., Frías-Cespedes M., Ainete M., Almirón W., Livdahl T. (2018). Modelling the distribution of the vector Aedes aegypti in a central Argentine city. Med. Vet. Entomol..

[9] Sommerfeld J., Kroeger A. (2013). Eco-bio-social research on dengue in Asia: A multicountry study on ecosystem and community-based approaches for the control of dengue vectors in urban and peri-urban Asia. Pathog. Glob. Health.

[10] Mitchell-Foster K., Beltran Ayala E., Breilh J., Spiegel J., Arichabala Wilches A., Ordóñez Leon T., Adrian Delgado J. (2015). Integrating participatory community mobilization processes to improve dengue prevention: An eco-bio-social scaling up of local success in Machala, Ecuador. Trans. R. Soc. Trop. Med. Hyg..

[11] San Martín J.L., Brathwaite Dick O. (2007). La estrategia de gestión integrada para la prevención y el control del dengue en la región de las Américas. Rev. Panam. Salud Pública.

[12] Usman H.B., Alsahafi A., Abdulrashid O., Mandoura N., Al Sharif K., Ibrahim A., Ahmed L., Shamrani E., Shamia M. (2018). Effect of health education on Dengue Fever: A comparison of knowledge, attitude, and practices in public and private high school children of Jeddah. Cureus.

[13] Ávila-Montes G.A., Martinez M., Sherman C., Fernández C.E. (2004). Evaluación de un módulo escolar sobre dengue y Aedes aegypti dirigido a escolares en Honduras. Rev. Panam. Salud Pública.

[14] Torres J.L., Ordóñez J.G., Vázquez-Martínez M.G. (2014). Conocimientos, actitudes y prácticas sobre el dengue en las escuelas primarias de Tapachula, Chiapas, México. Rev. Panam. Salud Pública.

[15] Escudero-Támara E., Villarreal-Amaris G. (2015). Intervención educativa para el control del dengue en entornos familiares en una comunidad de Colombia. Rev. Peru. Med. Exp. Salud Pública.

[16] Schweigmann N., Rizzotti A., Castiglia G., Gribaudo F., Marcos E., Burroni N., Freire G., D’Onofrio V., Oberlander S., Schillaci H. (2009). Información, conocimiento y percepción sobre el riesgo de contraer el dengue en Argentina: Dos experiencias de intervención para generar estrategias locales de control. Cad. Saúde Pública.

[17] Instituto Nacional de Estadística y Censos (INDEC) (2022). Resultados Provisionales del Censo Nacional de Población, Hogares y Viviendas 2022.

[18] (2019). Municipalidad de Córdoba. Córdoba una Ciudad en Cifras. https://gobiernoabierto.stage.cordoba.gob.ar/data/datos-abiertos/categoria/economia-y-finanzas/documento-cordoba-una-ciudad-en-cifras/13.

[19] Estallo E.L., Carbajo A.E., Grech M.G., Frías-Céspedes M., López L., Lanfri M.A., Ludueña-Almeida F.F., Almirón W.R. (2014). Spatio-temporal dynamics of dengue 2009 outbreak in Córdoba city, Argentina. Acta Trop..

[20] Ministerio de Salud de la Nación (MSN) (2009). Plan Nacional para la Prevención y el Control del Dengue y la Fiebre Amarilla. https://www.infoleg.gob.ar/basehome/actos_gobierno/actosdegobierno21-9-2009-1.htm.

[21] Gobierno de la Provincia de Córdoba (2020). Plan Estratégico de Abordaje Integral para la Prevención y el Control del Dengue y de la Chikungunya en Córdoba. https://www.cba.gov.ar/ministerio-educacion-dengue/.

[22] Alvarez Miño L., Taboada Montoya R. (2022). Manual de ciencia ciudadana. Primeros Pasos para Actuar Frente al Cambio Climático y la Salud.

[23] Shorten A., Smith J. (2017). Investigación con Métodos Mixtos: Ampliación de la Base de Evidencia.

[24] Okop K.J., Murphy K., Lambert E.V., Kedir K., Getachew H., Howe R., Berchmans Niyibizi J., Ntawuyirushintege S., Bavuma C., Rulisa S. (2021). Enfoque de ciencia ciudadana impulsado por la comunidad para explorar la percepción del riesgo de enfermedades cardiovasculares y desarrollar estrategias de promoción de la prevención en África subsahariana: Un protocolo de programa. Res. Involv. Engag..

[25] Haddawy P., Wettayakorn P., Nonthaleerak B., Yin M.S., Wiratsudakul A., Schöning J., Laosiritaworn Y., Balla K., Euaungkanakul S., Quengdaeng P. (2019). Large Scale Detailed Mapping of Dengue Vector Breeding Sites using Street View Images. PLoS Negl. Trop. Dis..

[26] Mahmud M.A.F., Mutalip M.H., Lodz N.A., Shahar H. (2018). Study on key Aedes spp breeding containers in dengue outbreak localities in Cheras district. Kuala Lumpur. Int. J. Mosq. Res..

[27] Viano L. Córdoba, Caso de Estudio para la Ciencia por el Dengue. La Voz. https://www.lavoz.com.ar/ciudadanos/cordoba-caso-de-estudio-para-ciencia-por-dengue/.

[28] Ferdousi F., Yoshimatsu S., Ma E., Sohel N., Wagatsuma Y. (2015). Identification of Essential Containers for Aedes Larval Breeding to Control Dengue in Dhaka, Bangladesh. Trop. Med. Health..

[29] Estallo E.L., Sippy R., Stewart-Ibarra A.M., Grech M.G., Benitez E.M., Ludueña-Almeida F.F., Ainete M., Frias-Cespedes M., Robert M., Romero M.M. (2020). A decade of arbovirus emergence in the temperate southern cone of South America: Dengue, Aedes aegypti and climate dynamics in Córdoba, Argentina. Heliyon.

[30] Muir L.E., Kay B.H. (1998). Aedes aegypti survival and dispersal estimated by mark-release-recapture in Northern Australia. Am. J. Trop. Med. Hyg..

[31] Weller S.C., Vickers B., Bernard H.R., Blackburn A.M., Borgatti S., Gravlee C.C., Johnson J.C. (2018). Open-ended interview questions and saturation. PLoS ONE.

[32] Brubacher S.P., Powell M., Skouteris H., Guadagno B. (2015). The effects of e-simulation interview training on teachers’ use of open-ended questions. Child Abuse Negl..

[33] Pan American Health Organization (PAHO) (2019). Technical Document for the Implementation of Interventions Based on Generic Operational Scenarios for Aedes Aegypti Control. https://iris.paho.org/bitstream/handle/10665.2/51652/9789275121108_eng.pdf?sequence=5&isAllowed=y.

[34] Di Rienzo J.A., Casanoves F., Balzarini M.G., Gonzalez L., Tablada M., Robledo C.W. (2020). InfoStat Version 2020. Centro de Transferencia InfoStat, FCA, Universidad Nacional de Córdoba, Argentina. http://www.infostat.com.ar.

[35] Braun V., Clarke V. (2006). Utilizando el análisis temático en psicología. Investig. Cual. En Psicol..

[36] World Health Organization (WHO) (2020). Dengue and Severe Dengue. https://www.who.int/news-room/fact-sheets/detail/dengue-and-severe-dengue.

[37] Ministerio de Salud de la Nación (MSN) (2020). Boletín Integrado de vigilancia N°507. https://bancos.salud.gob.ar/recurso/boletin-integrado-de-vigilancia-n507-se31-2020.

[38] Wilder-Smith A., Gubler D.J. (2008). Geographic expansion of dengue: The impact of international travel. Med. Clin. N. Am..

[39] Diaz-Quijano F.A., Martínez-Vega R.A., Rodriguez-Morales A.J., Rojas-Calero R.A., Luna-González M.L., Díaz-Quijano R.G. (2018). Association between the level of education and knowledge, attitudes and practices regarding dengue in the Caribbean region of Colombia. BMC Public Health.

[40] Abeyewickreme W., Wickremasinghe A.R., Karunatilake K., Sommerfeld J., Axel K. (2012). Community mobilization and household level waste management for dengue vector control in Gampaha district of Sri Lanka; an intervention study. Pathog. Glob. Health.

[41] Naing C., Ren W.Y., Man C.Y., Fern K.P., Qiqi C., Ning C.N., Ee C.W.S. (2011). Awareness of dengue and practice of dengue control among the semi-urban community: A cross sectional survey. J. Community Health.

[42] Quintero J., Carrasquilla G., Suárez R., González C., Olano V.A. (2009). An ecosystemic approach to evaluating ecological, socioeconomic and group dynamics affecting the prevalence of Aedes aegypti in two Colombian towns. Cad. Saúde Pública.

[43] Lun X., Yang R., Lin L., Wang Y., Wang J., Guo Y., Xu P., Zhu C., Liu Q., Xu L. (2023). Effects of the source of information and knowledge of dengue fever on the mosquito control behavior of residents of border areas of Yunnan, China. Parasites Vectors.

[44] Gregorio E.R., Takeuchi R., Hernandez P.M.R., Medina J.R., Kawamura S.Y., Salanguit M.B., Santillan M.D.C., Ramos K.M.S., Tuliao G.J., Morales L. (2024). Knowledge, attitudes, and practices related to dengue among public school teachers in a Central Luzon Province in the Philippines: An analytic cross-sectional study. Trop. Med. Health.

[45] Udayanga L., Gunathilaka N., Iqbal M.C.M., Pahalagedara K., Amarasinghe U.S., Abeyewickreme W. (2018). Socio-economic, Knowledge Attitude Practices (KAP), household related and demographic based appearance of non-dengue infected individuals in high dengue risk areas of Kandy District, Sri Lanka. BMC Infect. Dis..

[46] Alobuia W.M., Missikpode C., Aung M., Jolly P.E. (2015). Knowledge, Attitude, and Practices Regarding Vector-borne Diseases in Western Jamaica. Ann. Glob. Health..

[47] Toledo M.E., Baly A., Vanlerberghe V., Rodriguez M., Benitez J.R., Duvergel J., Van der Stuyft P. (2008). The unbearable lightness of technocratic efforts at dengue control. Trop. Med. Int. Health..

[48] Samsudin N.A., Othman H., Siau C.S., Zaini Z.I.I. (2024). Exploring community needs in combating aedes mosquitoes and dengue fever: A study with urban community in the recurrent hotspot area. BMC Public Health.

