# Supplementary material for: Empowering Communities through Citizen Science: Dengue Prevention in Córdoba
Source: Biology (Basel). 2024 Oct 15;13(10):826. doi: 10.3390/biology13100826 (PMC11504844; doi:10.3390/biology13100826)
Supplement: Supplementary file 1 [file biology-13-00826-s001.zip › biology-3201863-supplementary.pdf]

Empowering communities through citizen science: Dengue prevention in Córdoba

Table S1. Survey instrument including questions regarding the identification of vector-borne diseases, socio-ecological factors, knowledge, and perception of dengue, household vector prevention and control actions, as well as the household environmental surrounding.

Date:	School:
Class:	Name and surname of the student:
Name of the survey person:	Age:
Relationship with the student:<br>Mother Father Grandmother Grandfather Uncle<br>Aunt Sister Brother Other:	
Address:	
1) What is dengue?	
2) How is dengue transmitted?	
3) Do you know what *Aedes aegypti* is?	
4) Are all mosquitoes that bite Aedes aegypti?	
5) How do you know about dengue and Aedes aegypti?<br>Television ☐ Internet ☐ School ☐ Neighbours ☐ Friends ☐ Family members ☐<br>I didn´t receive information ☐ Other ☐	
6) For you, dengue is an illness:<br>Several ☐ Moderate ☐ Mild ☐ Don't know ☐	
7) Dengue prevention is for you a task that corresponds to<br>Each individual ☐ School ☐ Government ☐ Other: ☐	
8) Prevent on dengue believe it is<br>Easy ☐ Relatively difficult ☐ Impossible ☐ Don´t know ☐	
9) How many people live in your house?	
10) What activities do you do at home to avoid the presence of mosquitoes?<br>Cut the grass ☐ Turn container with water upside down and/ or eliminate them ☐<br>Add chemicals to the pool ☐ Use repellents or spirals ☐ Fumigation ☐ None ☐	
11) Do you know any case of dengue that has occurred in your neighbourhood?<br>Yes ☐ No ☐	
12) How many cases of dengue do you know that have occurred in your neighbourhood?	
13) Is there a garbage dump near your house? Yes ☐ No ☐	

14) How many meters away?
15) Is there a vacant lot near your house? Yes ☐ No ☐
16) How many meters away?
17) Is there a water channel near your house? Yes ☐ No ☐
18) How many meters away?
19) Is your house near the river? Yes ☐ No ☐
20) How many meters away?
21) Have you (or someone who lives with you) recently traveled to any of these areas?<br>North of the country ☐ Brazil ☐ Bolivia ☐ Paraguay ☐<br>Centroamérica/Caribe ☐ None ☐
22) When was the last time you (or that person) traveled to any of these places?
23) How often do you travel to these places?<br>Weekly ☐ Monthly ☐ Annual ☐ Other: ☐
24) In which months do you travel to those areas?
25) Do you have metallic screens on the windows in the home? Yes ☐ No ☐
26) Do you have metallic screens on the door in the home? Yes ☐ No ☐
27) Where do you see mosquitoes in your home?<br>Inside of the house ☐ Courtyard ☐ Garden ☐ Open gallery ☐ Other: ☐
28) How often do you see mosquitoes or larvae in your home?<br>Everyday ☐ Every week ☐ Other: ☐
29) Who is in charge of the care and maintenance of the green spaces in the house?
30) What kind of care does she/he perform?
31) Do you know the symptoms of dengue disease? Yes ☐ No ☐
32) What are the symptoms of dengue disease?
33) What is your highest degree of education?

34) Occupation:
35) Comments:

Table S2. Information provided by respondents on socio-ecological factors.

**Variable**	**Number**	**Percentage (%)**
Age		
18-29	21	11.2
≥30	167	88.9
Number of inhabitants for house		
1	1	1.0
2	19	9.8
3	32	16.5
**4**	**86**	**46.4**
5	35	18.6
≥ 6	15	7.7
Respondents with knowledge of dengue cases in their neighborhood		
Yes	17	9.0
No	171	91.0
Education level		
Primary	4	2.1
Secondary	62	33.0
**Tertiary**	**41**	**21.8**
**Graduate**	**71**	**37.8**
Postgraduate	8	4.3
Doesn´t answer	2	1.1
Recent travel destinations		
Don´t travel	150	79.8
**Brazil**	**25**	**13.3**
Caribbean/ Middle América	4	2.1
Bolivia	1	0.5
North of the country	13	6.9
Year of trips to risk areas		
**2020**	**39**	**43.8**
2019	20	22.5
2018	14	15.7
2017	5	5.6
<2016	11	12.4
Frequency of trips to risk areas		
Anual	83	97.6

Monthly	2	2.4
Months of trips to risk areas		
**January**	**51**	**27.1**
**February**	**30**	**16.0**
March	5	2.7
April	2	1.1
June	1	0.5
July	5	2.7
August	2	1.1
September	2	1.1
October	2	1.1
November<br>December	3<br>21	1.6<br>11.2

Table S3. Information provided by respondents on vector-borne diseases knowledge.

**Question**		**Responses**	**Number of responses**	**%**
What is dengue?				
	Correct	A disease	167	88.83
		An infection	4	2.13
		A virus	7	3.72
		TOTAL	178	94.68
	Not correct	A mosquito	9	4.79
		Doesn't know	1	0.53
		TOTAL	10	5.32
How is dengue spread?				
	Adequate	Through the bite of a mosquito	178	94.64
		The mosquito must have been infected with the virus	56	29.79
		The mosquito must first become infected (noting that not all mosquitoes are infected)	10	5.32
		The mosquitoes involved in transmission correspond to *Aedes aegypti*.	28	14.43

		Only the females who can transmit the virus	6	3.19
	Not correct	Sexually transmitted	1	0.53
		Blood transfusion	1	0.53
		Doesn't know	1	0.53
Do you know what Aedes aegypti *is?*				
	Adequate	A mosquito	137	72.68
		Transmitter of Dengue	103	54.79
		A transmitter of dengue and other diseases	14	7.45
		The species of the mosquito	16	8.51
		The scientific name	18	9.57
		Mention of some category in the style of taxonomy	37	19.70
	Not correct	Only " yes"	13	6.91
		Doesn't know	12	6.38
		The scientific name of the disease	3	1.6
		Dengue in its scientific term	1	0.53
		The infected mosquito called dengue	1	0.53
		The virus	1	0.53
Are all the biting mosquitoes Aedes aegypti*?*				
	Adequate	No	169	89.89
		The common mosquito too	1	0.53
	Not correct	Only females	2	1.06
		Yes	1	0.53
		Only infected mosquitoes	4	2.13

		Doesn't know	9	4.79
	TOTAL		188	

Table S4. Information provided by respondents on wha they know about dengue and *Aedes aegypti.*

	TV	INTERNET	SCHOOL	OTHER	n	%
	*				19	10.11
		*			20	10.64
			*		4	2.13
	*	*	*		26	13.83
	*	*			15	7.98
		*	*		22	11.70
	*			*	54	28.72
		*		*	5	2.66
			*	*	4	2.13
n	153	118	64	56		
%	81.38	62.77	34.04	29.79	0	0

Additional file S1: Additional information about teaching students.

Students were taught the definition of mosquitoes breeding sites, next to that urban environment such as our house and surroundings offer a wide variety of artificial containers that can contain water and could be mosquito breeding sites. They were shown that among the most common breeding sites in the city we can found: cans, jars, vases, buckets, bins, pet waterers, disused tires, toys, bottle caps, bag folds or disused canvas folds, pot plates, pots that do not drain water and leave accumulated water on their surface, drains where accumulated water can remain, any container that is not be using and that is exposed to rain.

The students learnt that the females mosquitoes use those containers that can contain water to lay their eggs and those sites are where part of the mosquito life cycle takes place. The eggs are placed on the wall of the artificial container, in the case of *Aedes aegypti* mosquitoes, vectors of viruses such as chikungunya, dengue and Zika. When the eggs are

covered with water, they hatch and give rise to the larvae develop which have an elongated shape, then they pass through the pupal stage and these develop into adults flying mosquitoes (female or male). Both females and males feed on plant sugars, but only the female feed on human and animals for blood whose proteins are useful for the development of its eggs.

On the other hand, students watched a YouTube video (https://www.youtube.com/watch?v=hTYkbgzbvQk&feature=youtu.be) about mosquito habitats, prevention, and dengue disease concepts. Besides, they read a local newspaper article with an interview conducted to our scientific research group in December 2019, about the dengue situation in the Americas during the end of 2019 and the warning expected situation for our city (https://www.lavoz.com.ar/ciudadanos/cordoba-caso-de-estudio-para-ciencia-por-dengue).

The students responded positively by putting into practice the knowledge acquired, identifying the types of containers they found, sending photos of them and clarifying whether or not they contained water, and whether mosquito larvae were observed.

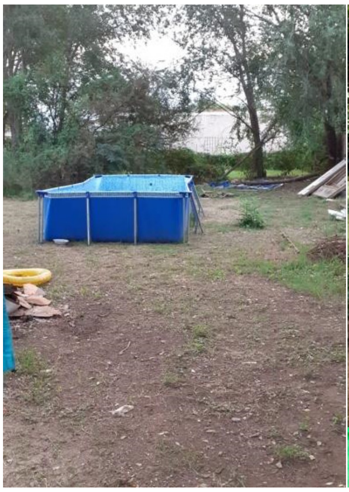

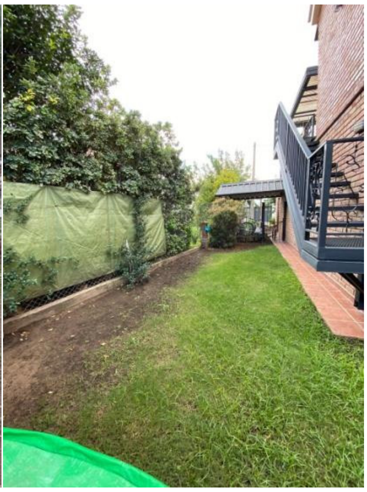

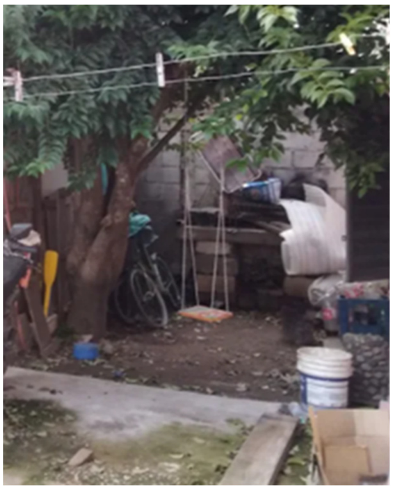

Photos taken by different students on their backyards and gardens.

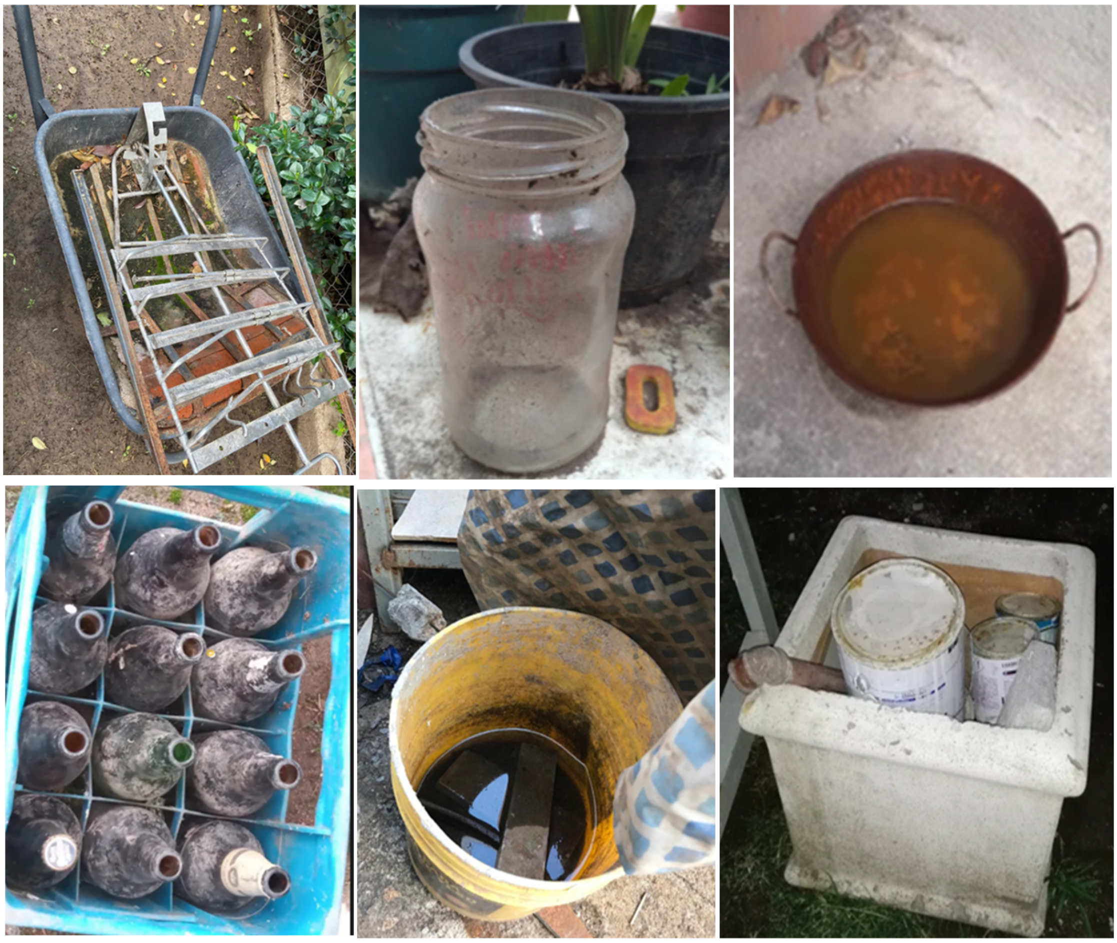

Some of the containers that students found with water and that spilled, or that could potentially contain water and then they turned them upside down or placed them out of the rain.

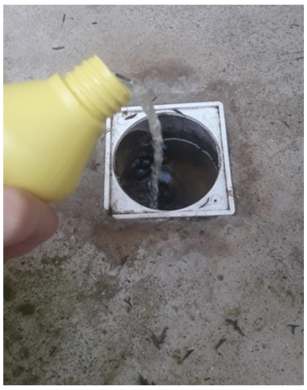

Students putting hypochlorite in the drains that found with water.

Finally, the students were asked to express an opinion about the activity carried out and some of which were:

“I believe that thanks to the activities, the text and the video, this disease will be taken more seriously, which we generally ignore, believing that it is an exaggeration or that it will never happen to us, but it is a serious problem that we are facing and if we all carry out the necessary care, this disease will stop affecting us.”

"I found that the recommendations and advice to eliminate mosquito eggs are easy to understand and carry out, and doing them each in your own home makes it very helpful to avoid and reduce the chances of contracting this disease."

“Very useful because we not only take care of ourselves but also the rest, it helps us to see clearly the importance of having this care. Preventing and taking care of our health”

"I think the activity is good because with this new information, for example, in my case, my brother knows more about dengue and its care, as do my parents when I tell him the information from the video and the news."

“This is good information since it allows us to learn more about the subject and train ourselves more about the dangers that we have in our surroundings and that sometimes we do not realise. These activities seemed very interesting and very interactive, as well as informative, now with all this information I am going to be more cautious.”
